# YLMY Tyrosine Residue within the Cytoplasmic Tail of Newcastle Disease Virus Fusion Protein Regulates Its Surface Expression to Modulate Viral Budding and Pathogenicity

**DOI:** 10.1128/spectrum.02173-21

**Published:** 2021-12-22

**Authors:** Yawen Bu, Qingyuan Teng, Delan Feng, Lu Sun, Jia Xue, Guozhong Zhang

**Affiliations:** a Key Laboratory of Animal Epidemiology of the Ministry of Agriculture, College of Veterinary Medicine, China Agricultural University, Beijing, China; University of Manitoba

**Keywords:** NDV, fusion protein, cytoplasmic tail, transporting and processing, cell surface, virus budding, virulence

## Abstract

Newcastle disease virus (NDV) fusion protein mediates the virus’s fusion activity, which is a determinant of NDV pathogenicity. The ectodomain of the F protein is known to have a major impact on fusion, and several reports have also indicated the role of the cytoplasmic tail (CT) in viral entry, F protein cleavage, and fusion, which are regulated by specific motifs. We found a highly conserved tyrosine residue located in the YLMY motif. The tyrosine residues at positions 524 and 527 have different roles in viral replication and pathogenicity and are associated with F protein intracellular processing. Tyrosine residues mutants affect the transportation of the F protein from the endoplasmic reticulum to the Golgi apparatus, resulting in different cleavage efficiencies. F protein is subsequently transported to the cell surface where it participates in viral budding, a process closely related to the distinctions in pathogenicity caused by the tyrosine residues. In addition, the different mutations all led to a hypofusogenic phenotype. We believe that the highly conserved tyrosine residue of the YLMY motif uses a similar mechanism to the tyrosine-based motif (YXXΦ) to regulate F protein transport and thus affect viral replication and pathogenicity.

**IMPORTANCE** The amino-terminal cytoplasmic domains of paramyxovirus fusion glycoproteins include trafficking signals that influence protein processing and cell surface expression. This study clarified that tyrosine residues at different positions in the YLMY motif in the cytoplasmic region of the F protein regulate F protein transportation, thereby affecting viral replication and pathogenicity. This study has increased our understanding of how NDV virulence is mediated by the F protein and provides a fresh perspective on the role of CT in the virus’s life cycle. This information may be useful in the development of NDV as an effective vaccine vector and oncolytic agent.

## INTRODUCTION

Newcastle disease (ND) is caused by the Newcastle disease virus (NDV), a highly epidemic and prevalent pathogen among avian species that causes high economic losses in worldwide poultry industries (1). NDV is a nonsegmented, single-stranded, negative-sense RNA virus (2). The two surface glycoproteins of NDV, the hemagglutinin-neuraminidase (HN) protein and the fusion (F) protein, interact with each other to accomplish viral entry (3). The F glycoprotein allows entry of the viral genome into the cytoplasm by fusing the viral membrane to the plasma membrane (4). Before participating in fusion, the F protein is initially synthesized by NDV as the inactive precursor F_0_, which has to be cleaved to become the disulfide-bonded F_1_ protein and F_2_ active complexed form (5). The F_1_ subunit contains two hydrophobic regions, the fusion peptide (FP), which resides at the new N-terminal after cleavage, and the transmembrane (TM) domain, which anchors the protein to the membrane of a virus or target cell, as well as two heptad repeat (HR) regions, HRA and HRB (6). All of these protein architectures are ectodomains, which are known to be important structural features of the F protein ectodomain. The FP initiates the process of fusion by penetrating the infected cell (7), triggering the HR1 and HR2 domains to undergo conformational changes that are reportedly necessarily for fusion (8). Thus, the FP may affect viral infectivity, replication, and pathogenicity (9). Several studies have shown that the TM domain also functions in protein folding, stability, and fusion (10, 11). Of these features belonging to ectodomains, the role of the cytoplasmic tail (CT) is the least well-understood.

The NDV fusion protein CT is 31 amino acids long, and as for other type I fusion glycoproteins, has been proven to play roles in regulating viral entry, F protein cleavage, fusion, and virion production. Mutational studies into human immunodeficiency virus type 1 (HIV-1), in which truncations and deletions of various lengths were introduced into the CT of gp41, have indicated that this region is important for infectivity and is associated with the incorporation of glycoproteins into virus particles (12–14). Deletion of the SARS-CoV-2 spike protein CT increased the virus’s infectivity in pseudovirus neutralization assays (15). Studies on other paramyxoviruses, including simian virus (SV5), parainfluenza virus 3 (PIV3), measles virus (MV), and parainfluenza virus 2 (PIV2), reported that the role of the F protein cytoplasmic domain in fusion varies with the F protein. Deletion of the entire domain from the PIV2 F protein and the MV F protein had no effect on cell surface expression and fusion activity, while F protein deletion from PIV3 affected protein folding and surface expression. In addition, the CT domain also influences the folding of the F protein ectodomain. Deletion of the carboxyl-terminal half of the NDV F protein cytoplasmic domain results in defective syncytium formation. The domain of the SV5 F protein is involved in later stages of fusion related to pore expansion (8, 16–20).

Paramyxovirus glycoproteins are synthesized in the endoplasmic reticulum (ER) and trafficked through the secretory pathway to the plasma membrane. Proper trafficking is needed for their incorporation into budding virions or fusion activity. Substantial amounts of evidence indicate CT has an important role in this process, and trafficking of viral glycoproteins has been demonstrated to involve certain motifs in CT, especially the Y-X-X-aliphatic/aromatic consensus motif. For instance, the CTs of Nipah (NiV) and Hendra (HeV) F proteins contain a YXXΦ motif that is required for the internalization of the protein by cells. NiV F proteins expressed from plasmid DNA are located primarily at the basolateral surface of epithelial cells, and this location depends on the YXXΦ motif in the CT of NiV F, and mutation of the Sendai virus TYTLE motif in the CT of the fusion protein deeply affects viral assembly and particle production (21–23). Additionally, such tyrosine-based motifs are often associated with endocytosis signals. Reportedly, replacement of tyrosine, analogous to the substitutions shown to abolish HIV polarized budding in epithelial cells, results in decreased endocytosis and the accumulation of lymphocytes at the surface of cells infected with either HIV-1 or simian immunodeficiency virus (24, 25). Most S proteins of alphacoronaviruses and gammacoronaviruses contain this motif in their CT. A previous study demonstrated that the YXXΦ motif is responsible for the intracellular retention but not the endocytosis of the TGEV S protein by cells (26, 27). The sorting signal is recognized by adaptor protein (AP) complexes (28–30), and selection of cargo proteins by the AP complexes requires the recognition of specific motifs found in the CT of transmembrane cargo. Recognition of the YXXΦ signals within retroviral structural proteins by AP2M1 and AP1M1 has been shown to be involved in mediating the intracellular trafficking of the Gag protein and infectious virus production and release (31). The YXXΦ motif also mediates HCV core binding to AP2M1 and HCV assembly (32, 33).

Through sequence alignment, we found a highly conserved tyrosine residue in the YLMY motif of the CT of the NDV F protein that is similar to a residue in the YXXΦ motif that has been previously demonstrated to be involved in fusion and apical and basolateral transport. In this study, we investigated the potential roles of the tyrosine residue of the YLMY motif in viral replication and pathogenicity. The effect of tyrosine residues on F protein intracellular processing was analyzed by an Endo H and glycopeptidase F digestion assays. By exploring the connection between F protein cell-surface expression and subsequent viral budding, we further clarified the specific mechanisms of how the tyrosine residue regulates viral replication and pathogenicity. This is a new discovery of a highly conserved tyrosine residue in the YLMY motif that also appears in known typical YXXΦ motifs yet regulates viral replication and pathogenicity by affecting F protein expression on the cell surface. Our findings have broadened our perspective on the mediation of NDV virulence by the F protein CT and provide a reference for other paramyxovirus studies.

## RESULTS

### Construction and rescue of YLMY motif tyrosine residue in mutant virus.

**FIG 1:**
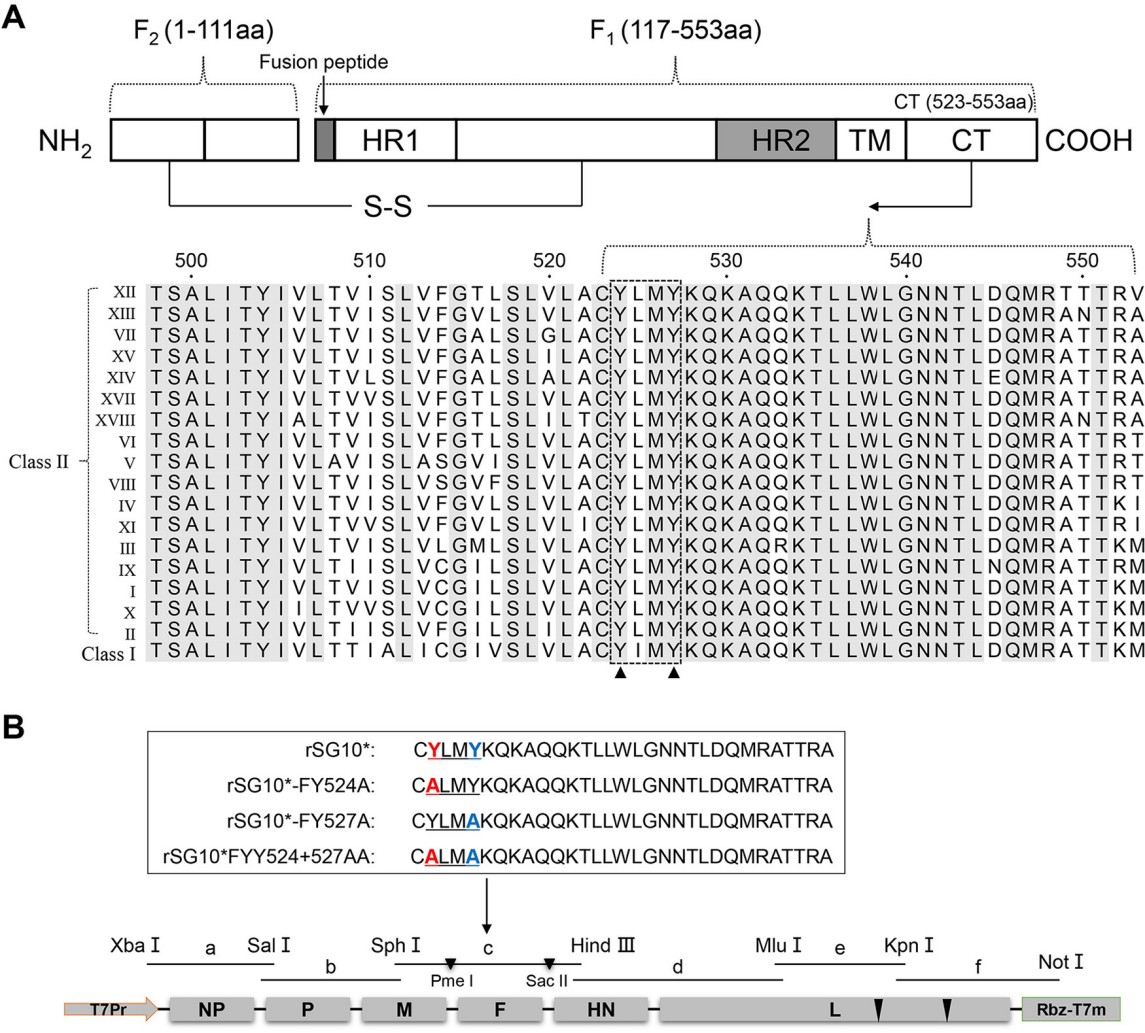
Construction and rescue of YLMY motif tyrosine residue mutant viruses. (A) Sequence alignment of F protein CT from different NDV genotypes. Putative key positions related to the function of the CT are marked with black arrowheads; strictly conserved residues are depicted with a gray background. (B) Schematic diagram of rSG10* and the location of mutations in the viruses. Tyrosine residues at different positions were mutated and marked in red or blue. The insertion position of the restriction site is indicated with a black arrowhead.

Sequence alignment of different NDV genotypes demonstrated that the YLMY motif is conserved between different NDV strains, with two tyrosine residues at positions 524 and 527 (Fig. 1A). Several viral membrane proteins have been proved to contain tyrosine (YXXΦ)-based targeting motifs. To verify the function of these Y motifs in the NDV life cycle, a progressive mutagenesis strategy was employed in which the Y-based motif was mutated along the F protein CT (Fig. 1B). The mutant viruses named rSG10*, rSG10*-FY524A, rSG10*-FY527A, and rSG10*-FYY524 + 527AA, were successively rescued using BSR T7/5 cells and embryonated eggs.

### YLMY motif tyrosine residue mutants show difference in viral replication and protein expression.

**FIG 2:**
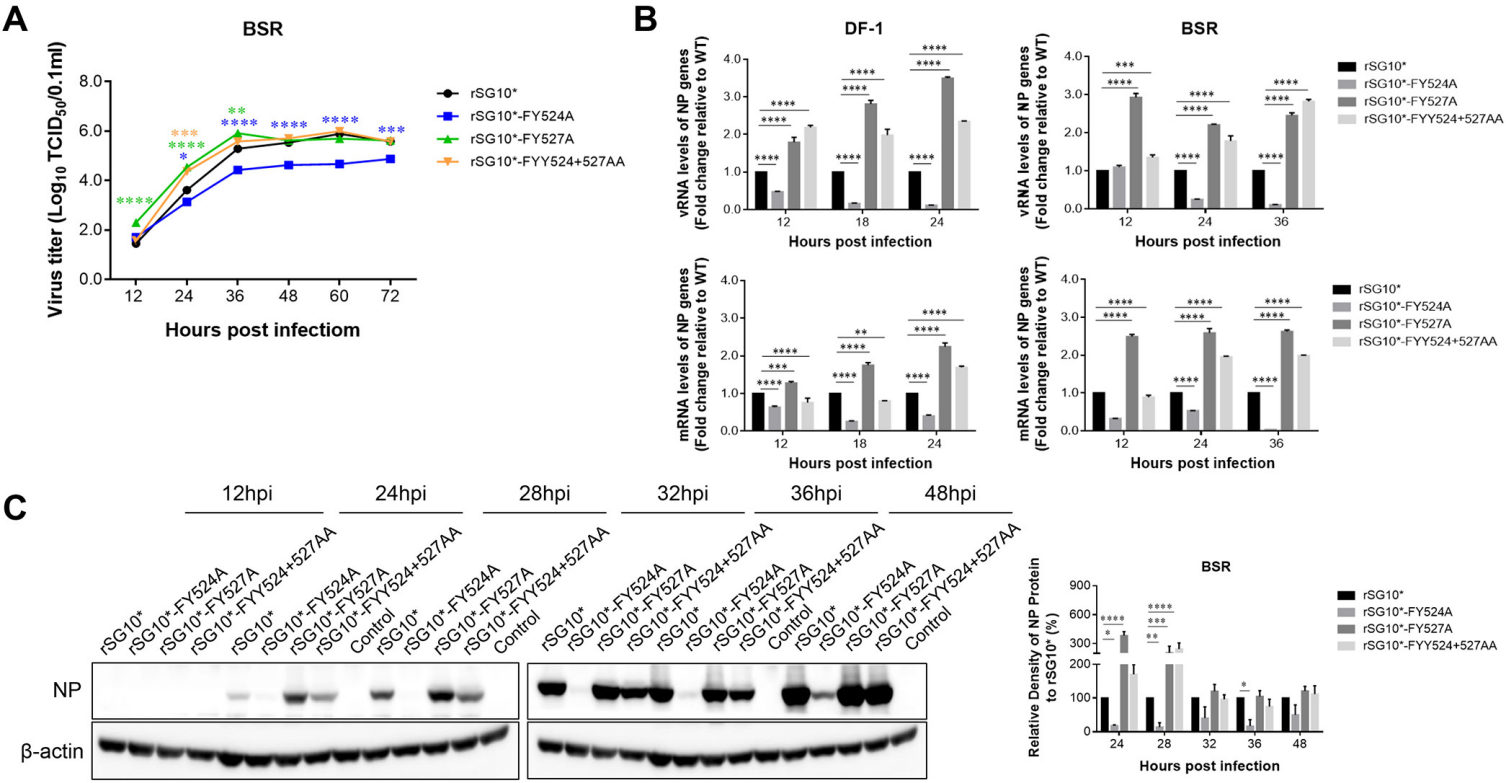
YLMY tyrosine residue plays an essential role in viral replication and protein expression. (A) Multistep (MOI = 0.01) growth curves of the four recombinant viruses in BSR-T7/5 cells. Asterisks indicate the significance of the difference between the recombinant viral titer and that of rSG10*. (B) Viral and mRNA synthesis in rSG10*- and YLMY-mutant infected cells. BSR-T7/5 or DF-1 cells were infected with different viruses at MOI = 0.1, and total RNA was purified from infected cells at the indicated times. Levels of RNA corresponding to the nucleoprotein segments were measured by quantitative real-time PCR. RNA levels were normalized to those of *GADPH*. (C) BSR-T7/5 cells were infected with different viruses at MOI = 0.1, The expression level of each NP protein was determined by Western blotting using anti-NP antibodies. NP protein expression levels are expressed as percentages of the levels for rSG10*, which were set at 100%. Scale bars represent 100 μm. *P values* were calculated by a two-way ANOVA. Statistical significance was set as follows: *, *P* = 0.01–0.05; **, *P* = 0.001–0.01; ***, *P* = 0.0001–0.001; *n* = 3.

The multistep growth kinetics of YLMY mutants were determined in BSR T7/5 cells (Fig. 2A). The single tyrosine mutant rSG10*-FY524A showed obviously defective replication during the entire infection process, while rSG10*-FY527A and dual-mutation rSG10*-FYY524 + 527AA replication was substantially faster than rSG_10_* until 36 hpi, after which the replication plateaued. We next investigated the RNA levels in those mutant viruses. Individually, the levels of genomic RNA and mRNA of rSG10*-FY527A and rSG10*-FYY524 + 527AA were persistently and significantly higher than those of rSG_10_*; however, those of rSG10*-FY524A were constantly at an extremely low level, and this phenomenon had no cell specificity (Fig. 2B). To further confirm whether there was a similar trend in viral protein expression, we next tested for NP protein expression during the different periods of viral infection. We found rSG10*-FY527A and rSG10*-FYY524 + 527AA possessed higher NP protein expression during the whole infection process, while that of rSG10*-FY524A remained at a low level (Fig. 2C). Together, these results demonstrated that the tyrosine 527 mutation significantly promoted viral replication and protein expression, while the tyrosine 524 mutation resulted in the opposite.

### YLMY motif tyrosine residue demonstrates adverse role in viral pathogenicity.

**FIG 3:**
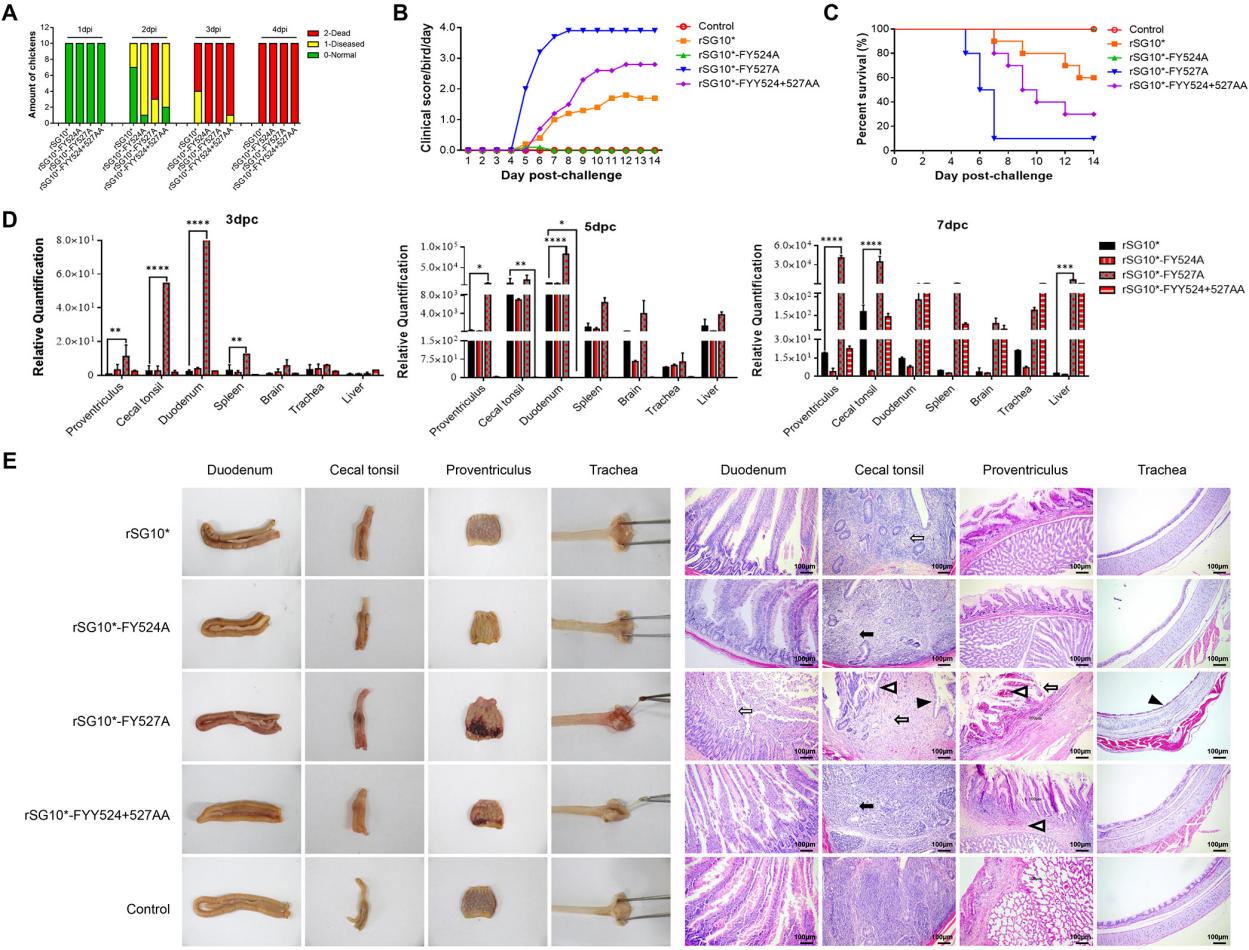
YLMY motif tyrosine residue mutants have adverse role in viral pathogenicity. (A) Specific pathogenesis during the ICPI observation. The average score was calculated according to the following criteria: 0, flexible activities and no phenomenon of ataxia; 1, paralyzed and lying on the ground, excepting sluggish chickens; 2, dead. (B) Clinical scores of rSG10* and YLMY mutant groups. Clinical signs were scored daily based on 10 birds per group (0, healthy; 1, sick; 2, wing drop/paralysis/torticollis/incoordination; 3, prostration; 4, dead). The daily mean scores for each group are shown. (C) Survival of 3-week-old SPF chickens inoculated with parental and chimeric viruses based on 10 birds per group. (D) Replications of rSG10* and mutants in 3-week-old chickens. The inoculated birds were sacrificed at 1, 3, 5, 7 dpi, the indicated tissues were collected, and virus loads determined by RT-PCR. (E) Gross lesions and tissue histopathology. Birds were sacrificed at 5 dpi, and tissues were fixed with formalin, sectioned, and stained with hematoxylin and eosin. The trachea had exfoliation of tracheal mucosa epithelium (black triangles). The duodenum had intestinal villi necrosis (white arrow). The cecal tonsil showed hemorrhage (white triangle), lymphocyte necrosis (white arrow), absence of lymphoid follicles (black arrow), and shedding of mucosal epithelial cells (black triangle). The proventriculus showed hemorrhaging (white triangle) and shedding of mucosal cells (white arrow).

We investigated the function of the YLMY motif in NDV pathogenesis using two standard pathogenicity assays, the mean death time (MDT) and the intracerebral pathogenicity index (ICPI). When we compared the mutant’s MDT, they were all ≤60 h, and the viruses had ICPI scores of between 1.5 and 2.0, which meant they were still virulent. The specific pathogenic features seen during the ICPI experiment are shown in a histogram in Figure 3A The virus titers showed that all mutants exhibited excellent reproductive performance in the embryos and BSR T7/5 cells. It is worth noting that rSG10*-FY527A achieved a higher virulence score and better virus titer compared with rSG_10_* and the other mutants (Table 1).

**TABLE 1:** Pathogenicity and virus titer of parental and YLMY motif tyrosine residue mutant viruses

Virus	Pathogenicity		Virus titer	
	MDT (h)^*a*^	ICPI score^*b*^	Log_10_ ELD_50_/ml	Log_10_ TCID_50_/ml
rSG10*	60.0	1.50	8.63	7.88
rSG10*-FY524A	57.6	1.61	8.56	7.95
rSG10*-FY527A	50.4	1.71	9.17	8.50
rSG10*-FYY524 + 527AA	57.6	1.58	9.17	8.00

a Mean death time (MDT) is the mean time for the minimum lethal dose of virus to kill all inoculated embryos. Pathotype: virulent strains, <60 h; intermediate virulent strains, 60 to 90 h; avirulent strains, >90 h.

b Intracerebral pathogenicity index (ICPI): velogenic strains,1.5–2.0; moderately virulent strains, 0.7–1.5; avirulent strains, 0.0 to 0.7. ELD_50_, median embryo lethal dose; TCID_50_, tissue culture infective dose.

We then further evaluated the pathogenicity of the mutants. All mutant viruses began to show clinical symptoms at 4 days postinoculation (dpi); the peak period for rSG10*-FY527A lasted from 4 dpi to 8 dpi, and the mortality rate was 90%; while rSG_10_* and rSG10*-FYY524 + 527AA exhibited a similar course of morbidity during 4 dpi to 7 dpi, but rSG10*-FYY524 + 527AA showed more severe clinical symptoms after 7 dpi. The mortality of rSG10* and rSG10*-FYY524 + 527AA were 40% and 70% respective. However, rSG10*-FY524A did not induce obvious clinical symptoms or mortality throughout the observation period, only slight depression during 4 dpi to 7 dpi (Fig. 3B and C). We tested the relative expression of total NP RNA to represent the amount of viral replication in the organs, and rSG10*-FY527A possessed an increased replication ability in many tissues during the sampling interval, especially in the target organ. Whereas rSG10*-FY524A presented a similar viral load to rSG10* in various organs at 3 dpc and 5 dpc but showed an obviously lower replication ability at 7 dpc in the target organ, while viral RNA was barely detected in the other organs. Contrastingly, infection with rSG10*-FYY524 + 527AA initially resulted in smaller amounts of virus in the various organs, but the mutant possessed a higher replication ability at 7 dpc compared with the parent virus, which is possibly why rSG10*-FYY524 + 527AA presented more severe clinical symptoms compared with rSG10* after 7 dpi (Fig. 3D). The necropsy and histology results showed that all viruses caused histological changes and tissue lesions comparable with the characteristics of the virulent strain, although rSG10*-FY527A and rSG10*-FYY524 + 527AA caused more serious damage than the other mutants and parent virus (Fig. 3E). These data demonstrated that the different tyrosine sites of YLMY play opposing roles in viral pathogenicity.

### YLMY motif tyrosine residue has no function in clathrin-mediated endocytosis.

**FIG 4:**
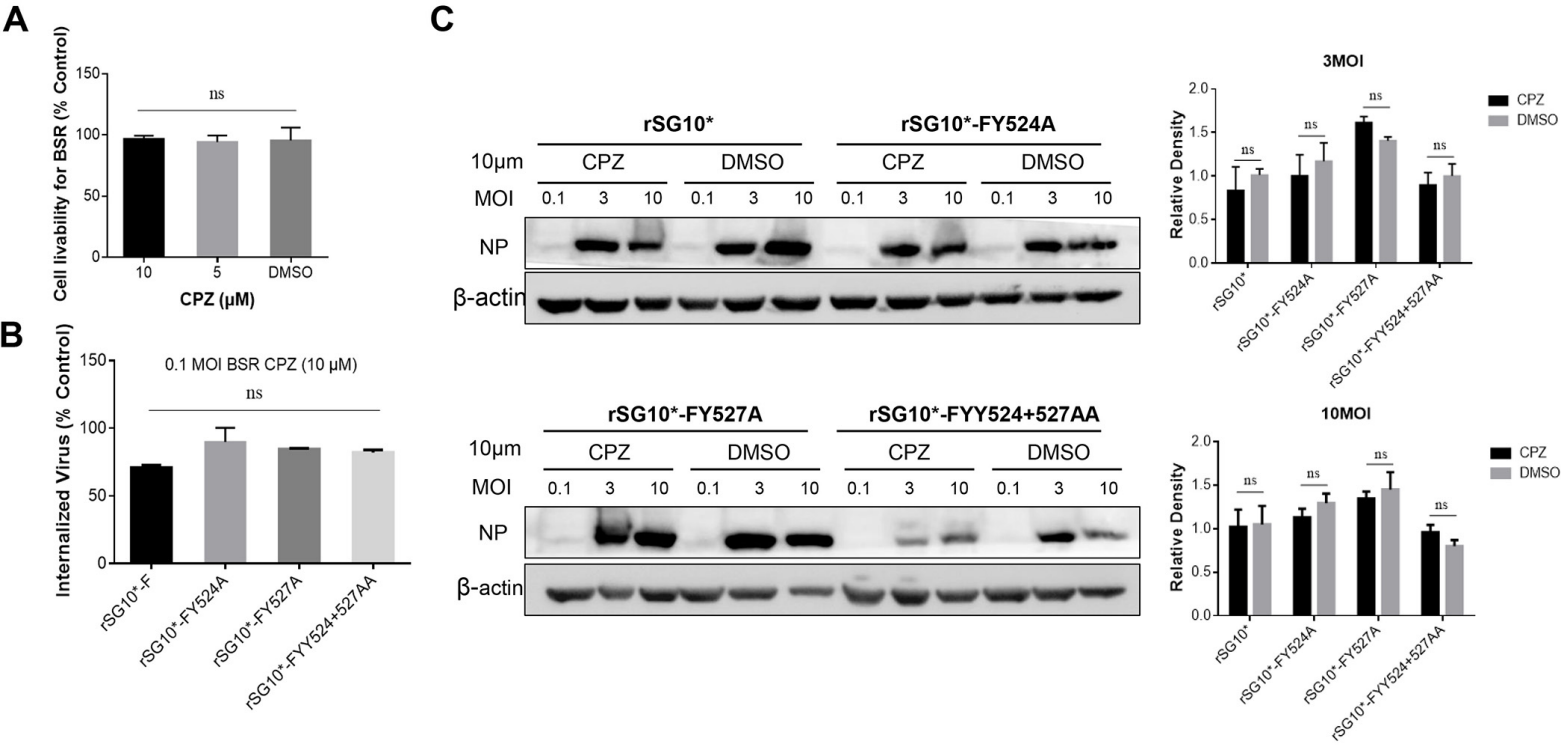
YLMY motif tyrosine residue does not function in clathrin-mediated endocytosis. (A) The cytotoxicity of chlorpromazine (CPZ). (B, C) BSR-T7/5 cells were pretreated with 10 μm CPZ at 37°C for 1 h and incubated with viruses for 1 h. DMSO was used as a negative control. The cells were collected at 6 hpi for qRT-PCR and Western blot to test the invasion efficiency of viruses. Results are presented as the mean ± SD of data from three independent experiments.

NDV can enter host cells through the fusion of its capsule membrane to the cytoplasmic membrane, but it can also use endocytosis to infect the host directly, especially clathrin-mediated endocytosis (CME), which largely depends on a tyrosine-based consensus motif present in the CT of the F protein. To identify whether changes in the YLMY motif affect NDV’s ability to use CME to enter cells, a specific chemical inhibitor (chlorpromazine, CPZ) was used to block the CME pathway. A cytotoxicity test showed that 10 μM CPZ had no effect on the viability of the BSR-T7/5 cells (Fig. 4A). The internalization rates of the mutants after CPZ treatment were quantified by qRT-PCR and Western blotting at 6 hpi. The qRT-PCR results showed that NDV invasion was inhibited by CPZ, by approximately 15%–25%, but there was no significant difference between the parental and mutant viruses (Fig. 4B). The same results were observed on the Western blot (Fig. 4C). Collectively, these data suggest that NDV can enter cells through the CME pathway, but the tyrosine residue of the YLMY motif does not function in CME.

### YLMY motif tyrosine residue mutants leads to discrepancies in F protein expression but confers hypofusogenic phenotypes.

**FIG 5:**
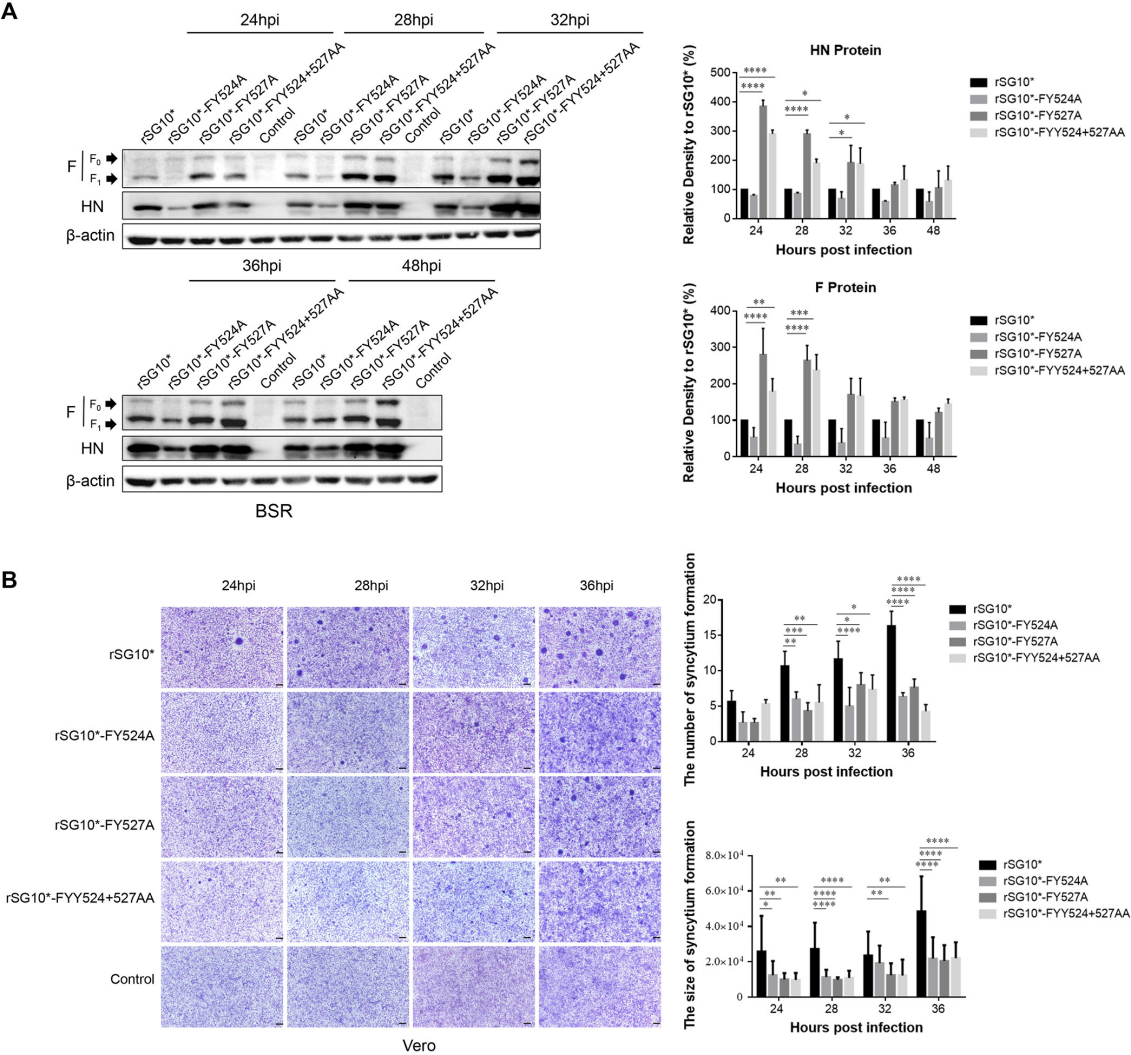
The expression and fusion activity of YLMY motif tyrosine residue mutants. (A) Western blot analysis of F YLMY mutants expressed in BSR-T7/5 cells. The cells were infected with virus at 0.1 MOI and cell lysates collected at the indicated times. The position of F_0_ and F_1_ are indicated by arrows in the left margins. (B) Conventional syncytium formation assay was induced by viral infection of Vero cells. After infection by mutant viruses, the cells were fixed with Giemsa solution. Syncytia were counted using a microscope. Each column and error bar represent the mean ± SD of syncytia for three independent experiments.

To investigate the effects of changes in tyrosine residues on F protein synthesis, the mutant viruses were used to infect BSR-T7/5 cells, and the results analyzed by Western blotting. The data show that F protein expression of rSG10*-FY527A and rSG10*-FYY524 + 527AA was higher than that of rSG_10_*, especially in the early stages of viral infection, while the same expression by rSG10*-FY524A was continuously lower than that of rSG_10_* during the whole infection. The same trend was observed for HN protein, meaning the function of the YLMY motif is not specific to the F protein (Fig. 5A). The F protein facilitates viral entry into cells by fusion. To investigate the significance of tyrosine changes in F-protein-mediated cell-cell fusion, we measured the fusion indices (number and size) of rSG_10_* and mutant viruses with Vero cells. The syncytia induced by the viruses with YLMY motif changes were markedly smaller and fewer (approximately 50% decrease, respectively) than those induced by rSG_10_* (Fig. 5B). This indicated that the tyrosine residues of the YLMY motif modulate F protein expression and cell-cell fusion.

### Intracellular processing of the F protein of YLMY motif tyrosine residues mutants.

**FIG 6:**
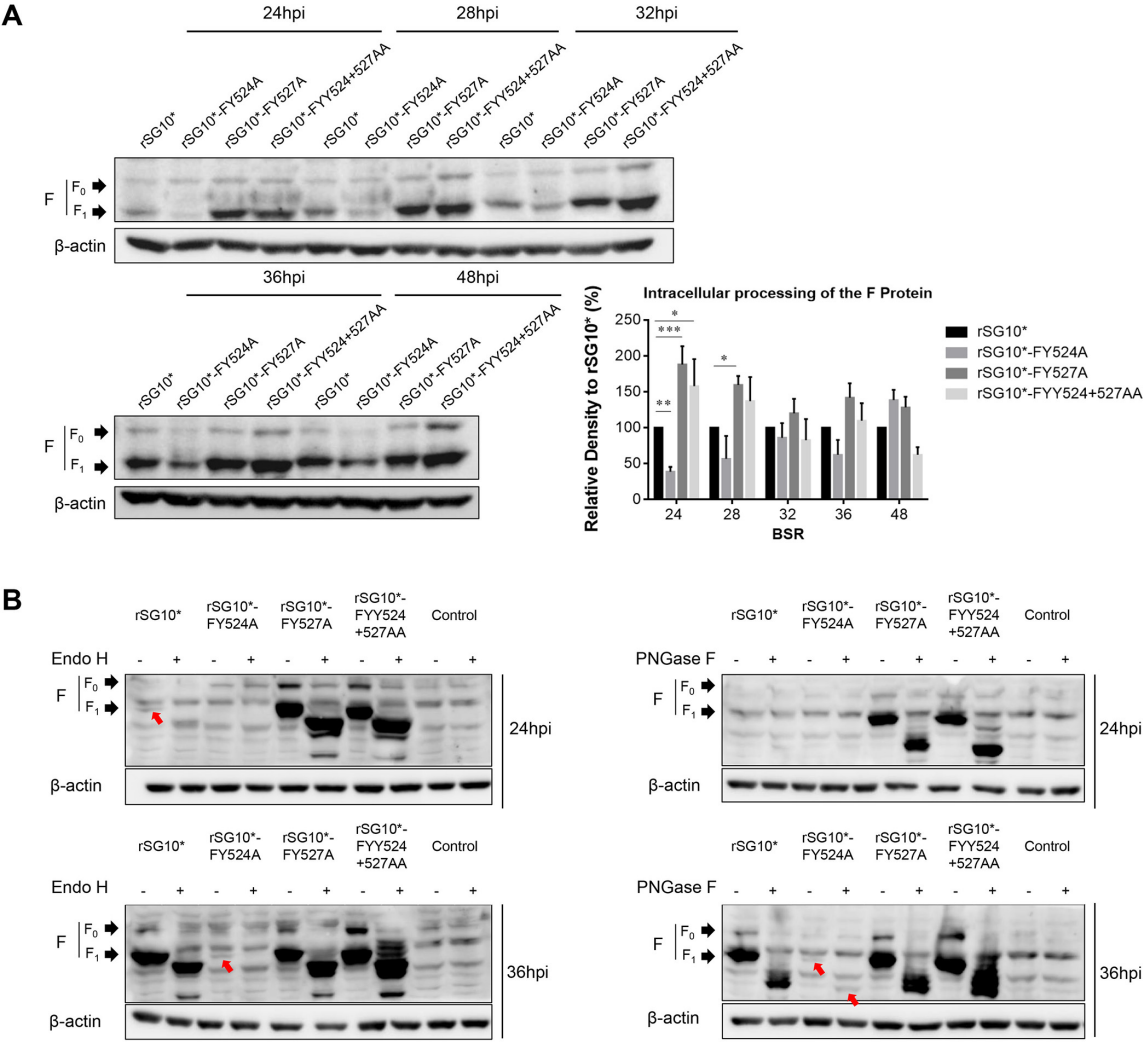
The intracellular processing of the F protein of YLMY motif tyrosine residue mutants. (A) The effect of critical tyrosine residues on F protein cleavage efficiency. The cleavage efficiency was measured by the ratio of F_1_ to F_0_. (B) Analysis of the difference in YLMY tyrosine residue mutant processing with Endo H and PNGase F digestion. Mutants were used to infect BSR-T7/5 cells, and protein was collected as described above. The next steps followed the Endo H and PNGase F kit instructions. Red arrows point to lighter F protein bands. Each column and error bar represent the mean ± SD for three independent experiments.

The NDV F protein is synthesized in the ER and then activated by proteolytic cleavage during transport within the Golgi organelle. Given the above results, we wanted to uncover whether and where those mutants have some discrepancy in F protein transportation and intracellular processing. The cleavage efficiency was measured by the ratio of F_1_ to F_0_. According to the results, the F_0_ protein was expressed and cleaved to F_1_-F_2_, but the extent of cleavage varied among the YLMY mutant viruses. In the rSG10*-FY527A and rSG10*-FYY524 + 527AA mutants, there was a higher percentage of F_1_/F_0,_ especially in the early infection stages, indicating the enhanced processivity of the F protein. While in the rSG10*-FY524A, both F_0_ and F_1_ were detectable; however, the cleavage efficiency (F_1_/F_0_) was lower than rSG10* (Fig. 6A). Given that this process happens in the Golgi, we used the Endo H enzyme, which can only digest the glycoprotein trapped in the ER and cannot digest protein in the Golgi, and peptide N-glycosidase F (PNGase F) to compare the glycan status of the mutants. The glycosylated precursors of all the virus genotypes shifted by a similar kDa of protein bands when treated with Endo H or PNGase F, suggesting that all the virus precursors contained similar protein modifications, but a difference in the processing time was seen. The results showed that, at 24 hpi, the Endo H-resistant protein band could still be detected for rSG10*-FY527A and rSG10*-FYY524 + 527AA mutants, while only the Endo H-sensitive protein band was recorded for rSG_10_*(red arrow). As for rSG10*-FY524A, there were no obvious bands detected. At 36 hpi, the Endo H-resistant Golgi processed mature F protein band was prominent in most viruses except for rSG10*-FY524A, which only showed a slight Endo H-sensitive protein band (red arrow, Fig. 6B). The results revealed that the tyrosine 527 mutant enabled the F protein to be rapidly transported to the Golgi, thereby promoting the processivity of the F protein, especially in the early infection stage. Whereas this process was obviously slower for the tyrosine 524 mutant.

### The F protein expression on the cell surface of YLMY motif tyrosine residue mutants.

**FIG 7:**
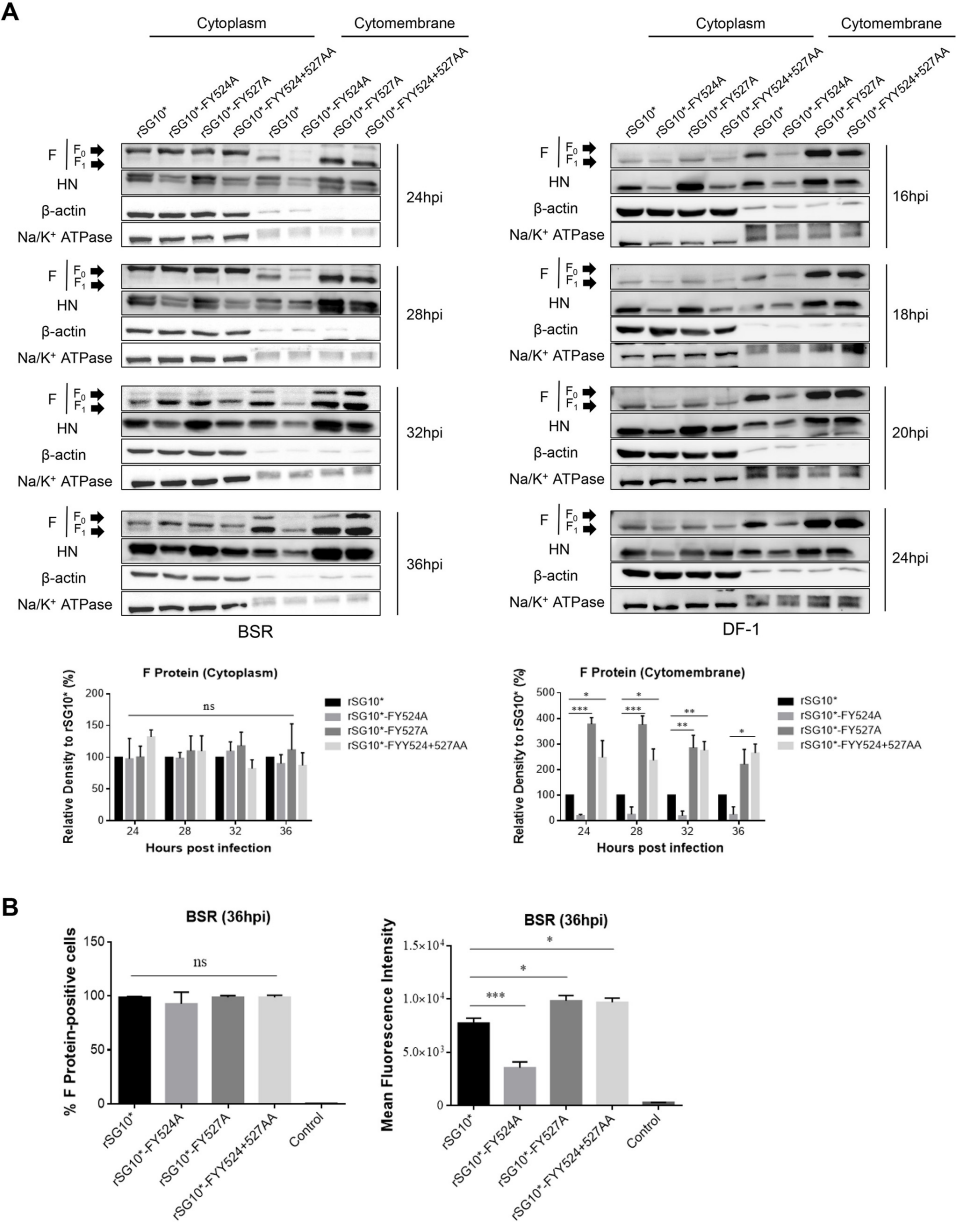
YLMY motif tyrosine residues mutants impact F protein expression on the cell surface. (A) Cell surface expression of F protein was determined by Western blotting. BSR-T7/5 and DF-1 cells were infected with each mutant virus at 0.1 MOI. The protein was extracted at 36 hpi following the kit instructions. (B) Cell surface expression of the F protein was determined by flow cytometry. BSR-T7/5 cells were infected with each mutant virus at 0.1 MOI. Surface expression of the F protein was assessed by flow cytometry at 36 hpi. Uninfected cells were used as negative controls. Values shown are the results from three independent experiments.

After synthesis in the ER and processing in the Golgi, the F protein is trafficked to the plasma membrane; thus, we wanted to understand the specific expression pattern of F protein on the cell surface. First, we detected the dynamic changes in F protein expression on the cell surface during infection using Western blotting. The results indicated that the F proteins of the mutants were all transported and assembled at the cell surface but to different degrees of success. The rSG10*-FY527A and rSG10*-FYY524 + 527AA mutants induced significantly more F protein expression at the cell surface compared with rSG_10_*, but the intracellular expression levels were similar. In comparison, the cell surface expression of the rSG10*-FY524A F protein was quite low (Fig. 7A). We next determined the level of F protein expression at the cellular membrane by flow cytometry. The virus infection efficiency was quantified as the percentage of F-positive cells, and F protein expression on the cell membrane was measured by the mean fluorescence intensity (MFI). The cell surface expression levels varied significantly among the YLMY motif mutants, despite the relatively constant level of F protein-positive cells, which is consistent with the trend shown by the Western blotting results (Fig. 7B). Together, these results show that tyrosine 527 mutant promotes the transportation of the intracellular synthesized F protein to the cell surface and its prominent expression, but this ability was weakened by tyrosine 524 mutant.

### The budding procession of the YLMY motif tyrosine residue mutants.

**FIG 8:**
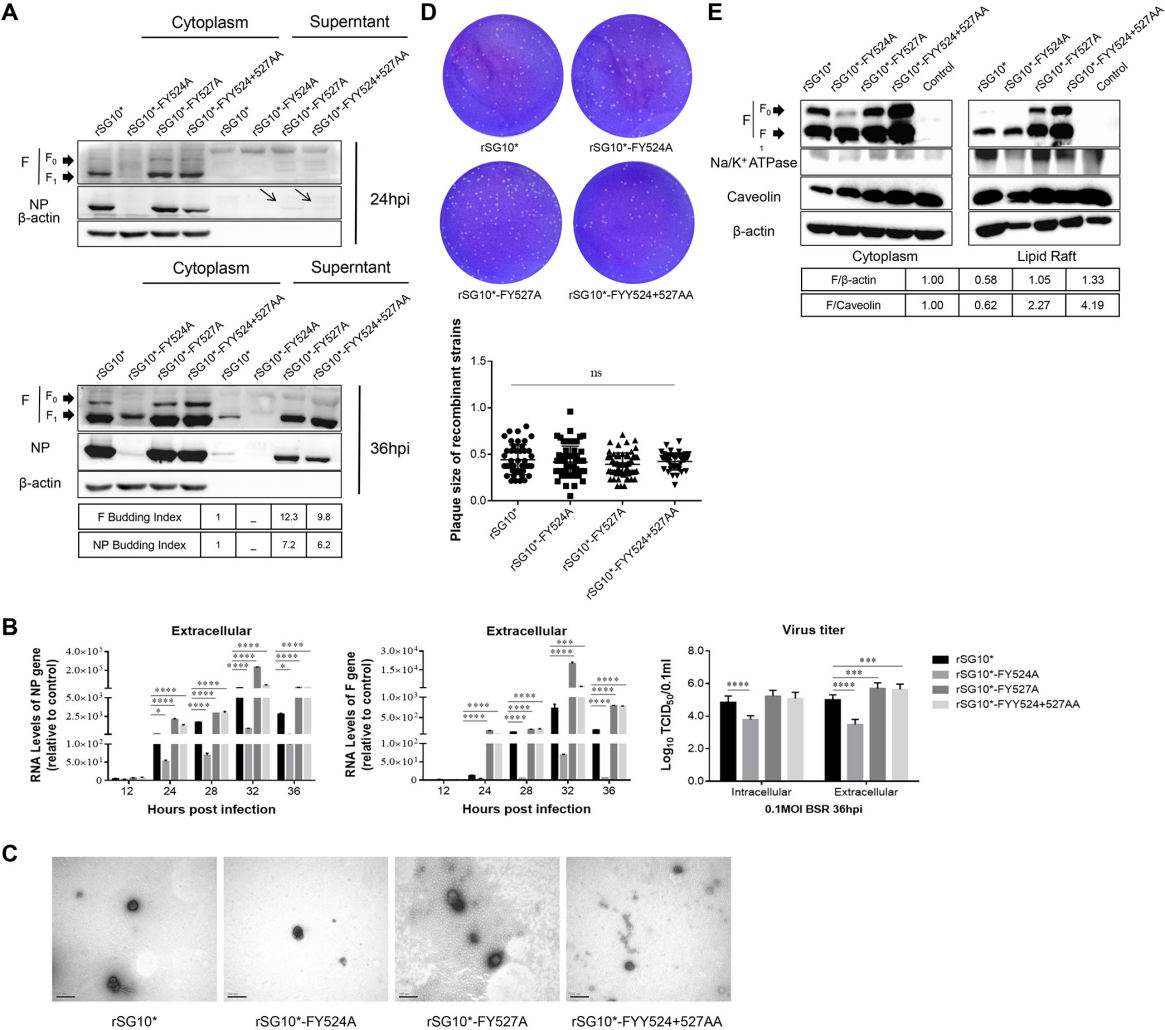
YLMY motif tyrosine residues affect virus budding. (A) BSR-T7/5 cells were infected with the rSG10* and mutant viruses for the indicated times. The cell lysates and viral particles were prepared and subjected to Western blot analysis. The band intensities were used to calculate budding indices, and all were normalized to rSG10*. (B) Intracellular and extracellular NP RNA expression. BSR-T7/5 cells were infected at 0.1 MOI, the supernatant was collected, and the cells washed with precooled PBS. The cells were separated by digesting with trypsin and collected using equal volumes of maintenance solution for further qRT-PCR and titer analyses. (C) Transmission electron micrograph analysis of particle release from BSR-T7/5 cells. (D) Plaque morphologies of rSG10* and YLMY mutants in BSR-T7/5 cells. Cells were infected with mutants and cultured at 37°C with medium containing agarose. Plaques were identified using crystal violet staining. *P values* were calculated based on a two-tailed, *unpaired t test* (95% confidence levels). (E) Colocalization of F protein and lipid rafts. BSR-T7/5 cells were infected with rSG10* and YLMY mutants at 0.1 MOI, and protein was collected at 36 hpi. Cell lysates were subjected to Western blotting with the indicated protein antibodies. The F protein of cell lysate and lipid rafts were used to calculate the relative expression, which was normalized to rSG10*.

Given the previous results, the different tyrosine sites of the YLMY motif mutants led to differences in the expression of F protein on the cell membrane. Previous pathogenicity experiments also proved that the position of tyrosine has different regulatory effects on virus pathogenicity. We speculated whether differences in F protein expression on the cell membrane surface regulate virus pathogenicity through some mechanism. Based on previous research, the viral glycoprotein is believed to cluster within lipid raft membranes to participate in budding, and the process is directed by its CT. We hypothesized that the YLMY motif mutants caused the difference in F protein expressed in the region of the cell surface where the lipid rafts are concentrated and subsequently participated in the virus budding process. To verify our assumptions, we first aimed to understand the role of the YLMY motif in virus budding. All viruses were assessed for whole-cell expression by Western blotting, and the levels of protein incorporated into viral particles were determined by sucrose cushion ultracentrifugation. The results showed that rSG10*-FY527A and rSG10*-FYY524 + 527AA had similar NP and F protein expression levels in the cell lysate to rSG_10_* but obviously higher levels in the supernatant. Owing to the low protein expression of rSG10*-FY524A, it was difficult to detect the bands corresponding to the virions, and we were not sure of the function of tyrosine 524 in viral budding (Fig. 8A). To rule out the effect of viral particle incompleteness, we tested the extracellular RNA levels of the NP and F proteins. A similar trend to protein expression was seen, and extracellular virus titers also demonstrated that the virus released into the supernatant existed in a complete and infectious form. Additionally, there were no obvious differences between rSG10*-FY527A, rSG10*-FYY524 + 527AA, and rSG_10_* in their intracellular virus titers, although rSG10*-FY527A and rSG10*-FYY524 + 527AA were present at significantly higher extracellular levels, which means that the tyrosine 527 mutation indeed promoted later viral budding. For rSG10*-FY524A, both the intracellular and extracellular virus titers were significantly lower than those of rSG10*; therefore, we were unsure of whether tyrosine 524 played any role in viral budding (Fig. 8B). To visualize the morphology of the virus particles, transmission electron microscopy (TEM) was carried out, and the mutants showed F protein spikes on their surface similar to those on rSG_10_*. Furthermore, the diameter of the viral particles was similar to those of rSG_10_* and was within the usual range for virions grown in other cell types (Fig. 8C). Combined with the above results, this indicated that viral budding was affected by the YLMY motif, especially the residue at position 527.

In addition to the release of virus particles into the supernatant, intercellular transmission is also a mechanism of viral spreading. To rule out the rSG10*-FY527A mutation as a cause of the enhanced viral budding at the cost of reduced intercellular transmission, we conducted a plaque experiment, and the results were in line with our expectations, i.e., there was no difference in the diameter of the plaques (Fig. 8D). Membrane lipid raft domains are thought to be sites of budding for NDV. Based on the above results, we speculated that the rSG10*-FY527A and rSG10*-FYY524 + 527AA mutants facilitate the transportation of the F protein to lipid rafts at the cell surface, which is beneficial for later viral budding. We used a kit designed to extract lipid rafts, and detected the amount of F protein expressed and bound to the rafts. The results showed no apparent F protein expression among the mutant viruses in whole cells. In terms of the amount of protein bound to the lipid rafts, rSG10*-FY527A and rSG10*-FYY524 + 527AA showed significantly more than rSG_10_*(Fig. 8E). Collectively, the tyrosine 527 of YLMY mutant promoted F protein transportation to the cell surface, thereby increasing the amount of F protein bound to the lipid rafts and promoting postviral infection budding, while tyrosine 524 weakened this capability.

## DISCUSSION

Several studies have demonstrated the roles of the CT of paramyxovirus glycoproteins in particle formation, membrane fusion, protein folding and oligomerization, and viral infectivity and replication. For NDV, mutations in the CT of the fusion glycoprotein depressed syncytium formation (34). It is believed that the role of the glycoprotein CT in paramyxoviruses depends on specific amino acid sequences or signals. Tyrosine-containing signals, especially YXXΦ in the CT of viral envelope glycoproteins, have been proven to be associated with those functions. Research has provided increasing evidence that mutagenesis of tyrosine motifs in the CT can influence viral fusion and infectivity (35–37). In this study, we described a tyrosine-like motif, YLMY, and discovered its role in NDV replication, infectivity, and protein transportation. We compared the CT of the F protein among different genotypes of NDV and demonstrated that the YLMY motif is highly conserved, especially the tyrosine residues at positions 524 and 527. We evaluated the effects of YLMY tyrosine mutation on NDV replication and pathogenesis, and unexpectedly, found that tyrosine residues at different positions in YLMY have contrary effects on viral replication and pathogenicity. The rSG10*-FY527A and rSG10*-FYY524 + 527AA virus significantly promoted viral replication, protein expression and pathogenicity, while the rSG10*-FY524A resulted in the opposite. In the pathogenicity experiment, rSG10*-FY524A presented a similar viral load to rSG10* at 3 dpc and 5dpc, but viral RNA was barely detected in the target and other organ at 7 dpc. This result might be a reasonable explanation why rSG10*-FY524A caused mild clinical symptoms and had a 100% survival rate. The commonly used vaccine strain La Sota could be detected only a few days after immunization just like rSG10*-FY524A, which means those viruses could not replicate well in the tissues. This difference also exists in bovine leukemia virus (BLV), which possesses three tyrosine motifs. Different motifs and positions are involved in unique regulation functions in syncytium formation, Env packaging, endocytosis, and membrane binding, which suggests this motif regulates viral lifecycles through different mechanisms (38).

The NDV F protein facilitates viral entry into cells by fusing the viral envelope with the host cell membrane. We further investigated the role of the YLMY motif in F protein expression and fusion. The effect of this motif on F protein expression was in line with the trends shown in viral NP expression, but all mutants showed a hypofusogenic phenotype, in contrast with the wild type (Fig. 5). Virus-mediated cell-cell fusion is an extremely complicated process, especially for some paramyxoviruses that require two distinct proteins. The attachment protein binds to the receptor, while the fusion glycoprotein needs to undergo conformational changes that drive virus-cell membrane fusion and viral entry. The mutation of tyrosine in the three YXXL sequences of the BLV transmembrane protein all resulted in the excessive expression of protein on the cell surface, but only the first two mutations conveyed an increased fusion ability, thus there was no direct correspondence between expression level and fusion ability. This phenomenon also can be found in the DIII-DI linker region of the NDV fusion protein; the L295A mutant showed almost abolished fusion activity, even though there was a similar level of cell surface expression. Mutations G377S, A378D, and L379A in the DI-DII linker of the F protein induced much larger syncytia than the wild type, but with F protein expression at the cell surface comparable to that of the wild type. A possible explanation is that this mutant may have had an influence on the interaction between HN and F (6, 39). Further experiments are needed to ascertain whether a specific mechanism exists through which the YLMY motifs regulate F-mediated fusion.

The NDV F protein is synthesized as a fusogenically inactive precursor form and requires proteolytic processing, the cleavage must occur prior to virus assembly (40). The YLMY motif mutants retained their ability to process F protein, but rSG10*-FY527A and rSG10*-FYY524 + 527AA demonstrated enhanced processivity and rSG10*-FY524A reduced processivity. According to the Endo H and glycosidase digestion results, the differences in cleavage ability were mainly associated with the transportation of the F protein to the Golgi; in rSG10*-FY527A and rSG10*-FYY524 + 527AA, the F protein was rapidly transported to the Golgi for subsequent cleavage, while rSG10*-FY524A presented obvious hysteresis (Fig. 6B). We concluded that this process was mainly affected by the dynamic F protein expression differences caused by the YLMY motif.

For some paramyxoviruses, specifically NDV, Mev and human parainfluenza viruses 2 and 3, data indicated that the fusion proteins co-interact following their synthesis in the ER and, thus, are transported to the cell surface as a metastable protein complex (41, 42). When we explored F protein expression on the cell surface, the unique expression patterns were presented for each mutation. There were no statistical differences in F protein intracellular expression between rSG_10_* and the mutants, but a significant difference in F expression on the membrane surface was observed. The YLMY motif thus regulates the transfer of F protein from the Golgi to the surface by some mechanism that is related to the position of tyrosine in the YLMY motif.

It is understandable that rSG10*-FY524A induced low levels of F expression on the cell surface and thus presented a hypofusogenic phenotype, but it is confusing that the rSG10*-FY527A and rSG10*-FYY524 + 527AA mutants both overexpressed F protein on the cell membrane and showed reduced fusion ability. Because of the roles of CT in viral budding, we tested the effects of the YLMY motif in the process, and we found that rSG10*-FY527A and rSG10*-FYY524 + 527AA significantly promoted budding and did not impair virus particle integrity. Lipid rafts are regions where virus particles are assembled and released, and we further uncovered that the rSG10*-FY527A and rSG10*-FYY524 + 527AA mutations led to a significantly increased F protein concentration at the lipid rafts, resulting in improved virus budding that was ultimately reflected in the pathogenicity. The influence of YLMY mutations on virus budding is mainly caused by the process of F protein transportation to the cell surface, but this does not exclude the action of YLMY on other factors to regulate F protein expression in areas of lipid raft distribution. Increasing evidence has demonstrated that the glycoprotein is needed to either recruit M to assembly or initiate budding. This process is well characterized for the SeV fusion protein, which depends on the TYTLE motif in the CT of the protein (23, 43). The host cytoskeleton has been shown to play an important role in budding with several paramyxoviruses, and mutations of some domains in F result in a significant reduction in SeV viral particles production, which is reminiscent of certain motifs of F proteins that bind to specific host proteins to participate in budding (44). The increased F-protein colocalization with lipid rafts may be another reasonable explanation for the hypofusogenic phenotype of the rSG10*-FY527A and rSG10*-FYY524 + 527AA mutants, as F proteins are concentrated where lipid rafts gather to participate in subsequent assembly and budding, leaving less F protein available to participate in cell fusion. The distribution of F protein expression at lipid rafts and other regions will need to be confirmed to prove our assumption.

We clarified the role of the tyrosine residue of the YLMY motif in the NDV life cycle (Table 2). Our results strongly indicate that the mediation of pathogenicity by the YLMY motif mainly depends on the position of tyrosine and associated with the expression on the cell surface. Deciphering the F protein intracellular trafficking mechanism and the signal that controls this will undoubtedly be important to our understanding of NDV pathogenesis, and the identification of sequences essential for viral transportation and release may form the basis for novel antiviral therapeutics. The roles of the F protein YLMY motif and the potential host binding factors in the transportation of F protein are of great interest. The highly conserved nature of the motif, coupled with its sensitivity to mutation, suggests that the YLMY motif located within CT of the F protein may conduct YxxΦ functions in order to subvert host protein sorting machinery, such as AP1 and AP2, and further facilitate NDV release, making it a subject of interest for further research. It will be interesting to identify potential antiviral targets common to both enveloped RNA and DNA viruses with the intention of preventing systemic infection.

**TABLE 2:** Effects of YLMY motif tyrosine residues discovered in this study^*a*^

YLMY functions	524 tyrosine	527 tyrosine
Protein expression	↓	↑
Syncytia formation	↓	↓
Intracellular processing	↓	↑
Surface expression	↓	↑
Colocalization with lipid raft	↓	↑
Budding	↓	↑
Pathogenicity	↓	↑

a Direction of black arrow represents the regulation effect: upward represents an increase, downward represents a decrease.

## MATERIALS AND METHODS

### Animal use and ethics statement.

All specific-pathogen-free (SPF) chickens and SPF embryonated eggs were purchased from Beijing Boehringer Ingelheim Vital Biotechnology Co., Ltd. (Beijing, China). All chickens were raised in isolators at China Agricultural University throughout the experiments, with feed and water provided *ad libitum*. The Beijing Administration Committee of Laboratory Animals approved the animal experimental protocols under the auspices of the Beijing Association for Science and Technology (approval ID SYXK [Jing] 2018-0038) and Ethical Censor Committee at China Agricultural University (CAU approval no. 20200195).

### Cell and viruses.

Baby hamster kidney (BHK-21) cells stably expressing T7 RNA polymerase (BSR-T7/5), an African green monkey kidney cell line (Vero), and a chicken embryo fibroblast cell line (DF-1) were all grown in Dulbecco’s modified Eagle’s medium (DMEM, Gibco, Grand Island, NY, USA) containing 10% fetal bovine serum (FBS, Gibco). The recombinant NDV strain rSG10 was generated in our laboratory.

### Construction of plasmids and recovery of mutant viruses.

YLMY motif mutant plasmids were individually inserted into the full-length antigenomic cDNA of strain rSG10 in place of the corresponding NDV F ORF by introducing the unique restriction enzyme sites *Pme* I and *Sac* II with the Seamless Assembly Cloning kit (Invitrogen, Carlsbad, CA, USA). Virus rescue was performed as described previously (45).

### Virus growth kinetics.

The growth kinetics of viruses were evaluated under multiple cycle growth conditions in BSR-T7/5 cells. Cells in 6-wells plates were infected with viruses at an MOI of 0.01. Supernatants were collected at 12 h intervals until 72 hpi, and the viral titers were quantified and expressed as median tissue culture infective doses (TCID_50_)/0.1 ml using the endpoint method (46).

### Quantification of RNA synthesis by quantitative RT-PCR.

BSR-T7/5 and DF-1 cells were collected from virus infection assays at the indicated times, and total RNA was extracted using the Cell Total RNA isolation kit (Foregene, Chengdu, China). The resulting RNA samples were reversed-transcribed using a previously reported method. Quantitative (q)RT-PCR assays were performed using M5 HiPer SYBR Primeix Estate (Mei5 Biotechnology, Beijing, China) in a LightCycle 96 (Roche, Basel, Switzerland), and gene expression was normalized to that of the housekeeping gene β-actin.

### Western blot.

Total protein lysates were extracted from infected or transfected cells with ice-cold RIPA lysis buffer. Cellular proteins were separated through 10% sodium dodecyl sulfate-polyacrylamide gel electrophoresis and transferred to a polyvinylidene difluoride (PVDF) membrane (Amersham Biosciences, Freiburg, Germany). Each PVDF membrane was blocked with 5% (wt/vol) skim milk and 0.1% Tween 20 in Tris-buffered saline (TBST) and incubated with a primary antibody at 4°C overnight. After being washed with TBST, the membranes were incubated with the corresponding horseradish peroxidase (HRP)-conjugated anti-chicken, anti-rabbit, or anti-mouse antibody for 1 h (Bioss Biotechnology, Beijing, China). The presence of HRP was detected using a Western Lightning chemiluminescence kit (CWBIO, Beijing, China). Protein bands were normalized to β-actin and quantified by densitometry using ImageJ software (National Institute of Mental Health, Bethesda, MD, USA).

### Plaque assay.

BSR-T7/5 cells were infected with viruses at an MOI of 0.001 in 6-well plates. After 1 h of adsorption, the inoculum was removed and replaced with an overlay medium containing 2% FBS and 1% agar. Following incubation for 5 days, the cells were washed with PBS and fixed with 4% formaldehyde for 6 h. Then 0.1% crystal violet staining solution was added, and images were taken after the cell layer had dried. Plaque sizes were measured using Image J (National Institutes of Health).

### Cytotoxicity and drug treatment.

To test the effects of inhibitors on mutant virus internalization, it is necessary to evaluate the cytotoxicity of the inhibitors on cells. The cells were seeded into 96-well plates, grown for 12 h, and treated with CPZ at the indicated concentration for 24 h. We used the CellTiter-Lumi Luminescent Cell Viability assay kit (Beyotime Biotechnology, Shanghai, China) to test the cell viability. To test the effect of CPZ on mutant virus internalization, the cells were pretreated with a certain concentration of CPZ for 1 h, then infected with mutant viruses at an MOI of 0.1 in the presence of drugs for 1 h. After washing with PBS, the cells were incubated in DMEM containing 2% FBS at 37°C for 6 h and collected for qRT-PCR and Western blot analyses.

### Cell surface expression of the mutant virus F proteins.

A cell membrane and cytoplasmic protein extraction kit (Beyotime Biotechnology) was used to analyze F protein expression at the cell membrane. Briefly, the cells was lysed and centrifuged by low-speed to remove the nucleus, and then obtained cell membrane precipitation and cell supernatant contained cytoprotein, and then using membrane protein extraction reagents to obtain membrane protein, which not only includes cell membrane, but also the mitochondrial, the endoplasmic reticulum and the golgi apparatus membrane. For quantification of F protein cell surface expression levels by flow cytometry, BSR-T7/5 cells were infected with viruses at an MOI of 0.1; then at 36 hpi, the infected cells were digested and centrifuged at 800 × *g* for 5 min at 4°C. The cells were then incubated with rabbit anti-F antiserum (1:20 dilution) for 1 h at 4°C. Subsequently, the cells were washed three times with PBS, incubated for 1 h at 4°C with 1:50 diluted FITC-conjugated goat anti-rabbit immunoglobulin G antibodies, and analyzed using BD FACSCanto II. The percentage of F-positive cells and MFI were analyzed by FlowJo software.

### Glycopeptidase F and Endo H digestion.

For glycopeptidase F digestion, total protein lysates were extracted from infected cells following the Western blotting steps above. The protein was incubated for 10 min at 100°C, then incubated with 1% NP-40 and 1,000 U of glycopeptidase F (New England Biolabs, Beijing, China) in a total volume of 25 μl for 1 h at 37°C. For endoglycosides H (Endo H) digestion, the protein was adjusted to glycoprotein denaturing buffer and incubated for 10 min at 100°C, and we then added 10 × Glycobuffer 3 and Endo H (New England Biolabs) followed by incubation for 1 h at 37°C. The next steps were as described for the Western blotting.

### Fusion assessment.

The fusogenic abilities of the mutant viruses were examined using Vero cells. The cells were seeded into 12-well plates and infected with recombinant viruses at an MOI of 0.1. At various time points, we detected the dynamic changes in the size and number of syncytia. The specific operational steps were as follows: the cells were washed with PBS, fixed in methanol for 20 min at room temperature, and stained with Giemsa.

### Virus budding assay.

BSR-T7/5 cells were grown to 90% confluence and infected with the mutant viruses at an MOI of 0.1. At 36 dpi, the culture medium was collected and centrifuged at 5000 × *g* for 15 min to remove cell debris, then layered onto a cushion of 20% (wt/vol) sucrose in PBS, and subsequently ultracentrifuged in a SW41 Beckman centrifuge tube at 40,000 rpm for 2 h at 4°C. The viral particles pelleted at the bottom of the tubes were resuspended in STE (10 mM Tris-HCL, 100 mM Nacl, 1 mM EDTA) buffer. Samples were boiled and analyzed by Western blotting as described above. The amounts of protein in the cell lysates and viral particles were estimated based on the density of the protein bands using Image J software, and the budding index was calculated as follows: the amount of protein in viral particles/the amount of protein in the corresponding lysates, both normalized to the values obtained with rSG_10_* protein, which were set at 100%.

### Lipid raft association with F protein.

Cell monolayers were washed with PBS, and lipid rafts were extracted using the Minute Plasma Membrane-Derived Lipid Raft isolation kit (Invent Biotechnologies, Eden Prairie, MN, USA). The lipid raft concentration was determined with a bicinchoninic acid protein assay kit (CWBIO, Beijing, China). The cellular proteins were separated with 10% SDS-PAGE and detected by Western blot analysis.

### Transmission electron microscopy.

To prepare the virions for TEM, BSR-T7/5 cells were cultured in 6-well plates and infected with YLMY mutants at an MOI of 0.1. The culture medium was collected after 36 h, and virions were purified by ultracentrifugation through a 20% sucrose cushion. Samples were centrifuged at 28,000 rpm for 2 h; pellets were resuspended in 3 ml of STE buffer; and layers containing 20% sucrose (3 ml), 35% sucrose (3 ml), and 50% sucrose (3 ml) were applied to the tops of the samples, which were then centrifuged at 28,000 rpm for 2 h. A sample was collected from 35%–50% of the liquid, and PBS was added to dilute the purified virus, which was then centrifuged as previously. The final pellets were resuspended in 100 μl of ultrapure water; samples were absorbed onto a carbon-coated copper grid, negatively stained with 1% phosphotungstic acid (pH 7.0), and analyzed under a transmission electron microscope.

### MDT and ICPI of mutant viruses.

The virulences of the chimeric viruses were determined with standard virulence tests for NDV: the MDT in 9-day-old SPF embryonated chicken eggs and the ICPI in 1-day-old chickens. All tests were performed according to previously published methods (46).

### Pathogenicity assessment in chickens.

To evaluate the pathogenicity of the mutant viruses, groups of 20 (10 for sampling and 10 for clinical observation) 3-week-old SPF chickens were inoculated with 10^4.0^ 50% egg lethal dose (ELD_50_) of viruses per bird via the oculonasal route. The birds were observed daily and scored as follows for clinical signs at 14 dpi: 0, healthy; 1, sick; 2, wing drop/paralysis/torticollis/incoordination; 3, prostrated; 4, dead. Survival was monitored until 14 dpi. Two birds from each group were euthanized at 1, 3, 5, and 7 dpi, and the brain, trachea, lung, spleen, duodenum, and cecum were collected for virus titration and histopathology. The virus titers were determined by qRT-PCR as described above. For histopathology, fixed tissues were routinely embedded in paraffin wax, and 5-μm thick sections were prepared for hematoxylin and eosin staining and examined for lesions using light microscopy.

### Data analysis.

All data were analyzed using Prism 6.0 (GraphPad Software Inc., San Diego, CA, USA). Statistical differences among different groups were assessed using the analysis of variance method followed by Tukey’s test. Statistical significance was set at ∗*P* < 0.05, ∗∗*P* < 0.01, and ∗∗∗*P* < 0.001.
